# Lithium Sensitive ORAI1 Expression, Store Operated Ca^2+^ Entry and Suicidal Death of Neurons in Chorea-Acanthocytosis

**DOI:** 10.1038/s41598-017-06451-1

**Published:** 2017-07-25

**Authors:** Lisann Pelzl, Stefan Hauser, Bhaeldin Elsir, Basma Sukkar, Itishri Sahu, Yogesh Singh, Philip Höflinger, Rosi Bissinger, Mohamed Jemaà, Christos Stournaras, Ludger Schöls, Florian Lang

**Affiliations:** 1 Department of Physiology I, University of Tübingen, Tübingen, Germany; 2 German Center for Neurodegenerative Diseases, Tübingen, Germany; 3 Department of Biochemistry, University of Crete Medical School, Heraklion, Greece; 4 Department of Neurology and Hertie Institute for Clinical Brain Research, University of Tübingen, Tübingen, Germany

## Abstract

Chorea-Acanthocytosis (ChAc), a neurodegenerative disorder, results from loss-of-function-mutations of chorein-encoding gene VPS13A. In tumour cells chorein up-regulates ORAI1, a Ca^2+^-channel accomplishing store operated Ca^2+^-entry (SOCE) upon stimulation by STIM1. Furthermore SOCE could be up-regulated by lithium. The present study explored whether SOCE impacts on neuron apoptosis. Cortical neurons were differentiated from induced pluripotent stem cells generated from fibroblasts of ChAc patients and healthy volunteers. ORAI1 and STIM1 transcript levels and protein abundance were estimated from qRT-PCR and Western blotting, respectively, cytosolic Ca^2+^-activity ([Ca^2+^]_i_) from Fura-2-fluorescence, as well as apoptosis from annexin-V-binding and propidium-iodide uptake determined by flow cytometry. As a result, ORAI1 and STIM1 transcript levels and protein abundance and SOCE were significantly smaller and the percentage apoptotic cells significantly higher in ChAc neurons than in control neurons. Lithium treatment (2 mM, 24 hours) increased significantly ORAI1 and STIM1 transcript levels and protein abundance, an effect reversed by inhibition of Serum & Glucocorticoid inducible Kinase 1. ORAI1 blocker 2-APB (50 µM, 24 hours) significantly decreased SOCE, markedly increased apoptosis and abrogated the anti-apoptotic effect of lithium. In conclusion, enhanced neuronal apoptosis in ChAc at least partially results from decreased ORAI1 expression and SOCE, which could be reversed by lithium treatment.

## Introduction

Chorein promotes activation of phosphoinositide-3-kinase (PI3K)-p85-subunit and thus participates in the regulation of actin polymerization and cell survival^1–3^. Loss-of-function-mutations of the chorein encoding gene VPS13A (vacuolar protein sorting-associated protein 13A) lead to chorea-acanthocytosis (ChAc)^4, 5^, a progressive autosomal recessive neurodegenerative disease with hyperkinetic movements, impaired cognitive functions, myopathy with increased plasma levels of creatine kinase, and erythrocyte acanthocytosis^4, 6–8^. The movement and motor disorders of chorea-acanthocytosis include limb chorea, parkinsonism, dystonia, tongue protrusion, dysarthria, dysphagia, tongue and lip biting as well as progressive distal muscle wasting and weakness^8^.The derangements of motor function are paralleled by cognitive impairment, behavioral changes and epileptic seizures^8^. The progressive neurodegeneration eventually leads to severe disability and early death^8^. Gene targeted mice lacking functional chorein display erythrocyte shape changes^9^, neuronal apoptosis^10^ and altered behaviour^10^.

Chorein is expressed in a wide variety of tissues^11–13^ and participates in the regulation of diverse functions including dopamine release^14^, platelet activation^13^, cytoskeletal architecture^15^, endothelial cell stiffness^12^, and tumour cell survival^3^. Most importantly, chorein is decisive for the survival of neurons and skeletal muscle cells^4, 16^.

Regulators of cell survival and cell death include alterations of cytosolic Ca^2+^ activity ([Ca^2+^]_i_)^17, 18^. [Ca^2+^]_i_ could be increased by Ca^2+^ release from intracellular stores with subsequent store-operated Ca^2+^ entry (SOCE) through the pore-forming Ca^2+^ channel subunits ORAI1, ORAI2 and/or ORAI3^19^. Following store depletion, the ORAI isoforms are activated by the Ca^2+^ sensing proteins STIM1 and/or STIM2^20–22^. ORAI1 and SOCE are up-regulated by the PI3K pathway and thus at least in some cell types sensitive to chorein^23^. Moreover, ORAI1 and SOCE could be up-regulated by lithium^24^, which has been shown to counteract neurodegeneration^25–27^.

The present study explored whether chorein deficiency and lithium impact on neuronal ORAI1 expression, SOCE and/or cell survival. To this end skin fibroblasts from ChAc patients and age-matched healthy individuals were reprogrammed and differentiated to neurons and ORAI transcript levels, ORAI protein abundance, SOCE and apoptosis determined without or with prior lithium treatment.

## Results

In order to test whether the pathophysiology of chorea-acanthocytosis (ChAc) involves deranged neuronal regulation of the Ca^2+^ release activated Ca^2+^ channel ORAI1 and/or its regulator STIM1, experiments were performed with neurons generated from induced pluripotent stem cells (iPSCs). The iPSCs were generated from skin fibroblasts of healthy individuals (control neurons) and patients with chorea-acanthocytosis (ChAc neurons).

### ORAI1 and STIM1 transcript levels and protein abundance

**Figure 1:**
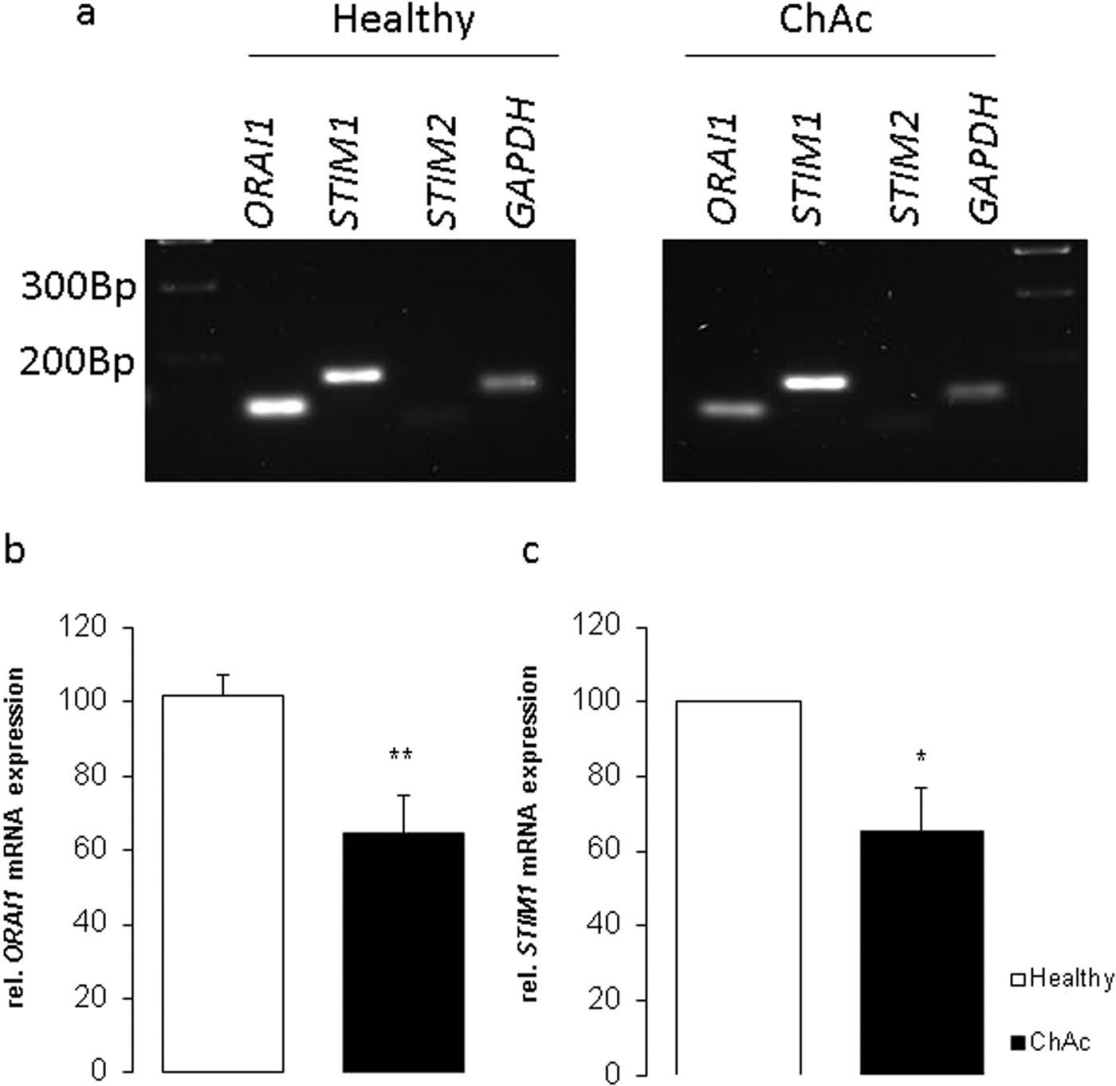
ORAI1 and STIM1 transcript levels in neurons generated from ChAc patients and healthy volunteers. (**a**) Original gels illustrating specificity of PCR products. (**b**,**c**) Arithmetic means (±SEM, n = 4) of (**b**) ORAI1 and (**c**) STIM1 transcript levels in neurons isolated from healthy volunteers (white bars) or from ChAc patients (black bars). *(p < 0.05), **(p < 0.01) indicates statistically significant difference to respective value from healthy volunteers.

qRT-PCR was utilized for the quantification of the transcript levels encoding ORAI1 or STIM1. As apparent from Fig. 1a,b, the neurons from both, healthy individuals and ChAc patients did express ORAI1 and STIM1. The ORAI1 and STIM1 transcript levels were significantly lower in neurons derived from ChAc patients than in neurons derived from healthy individuals  ﻿(Complete gel picture in Supplementary Figure S1).

**Figure 2:**
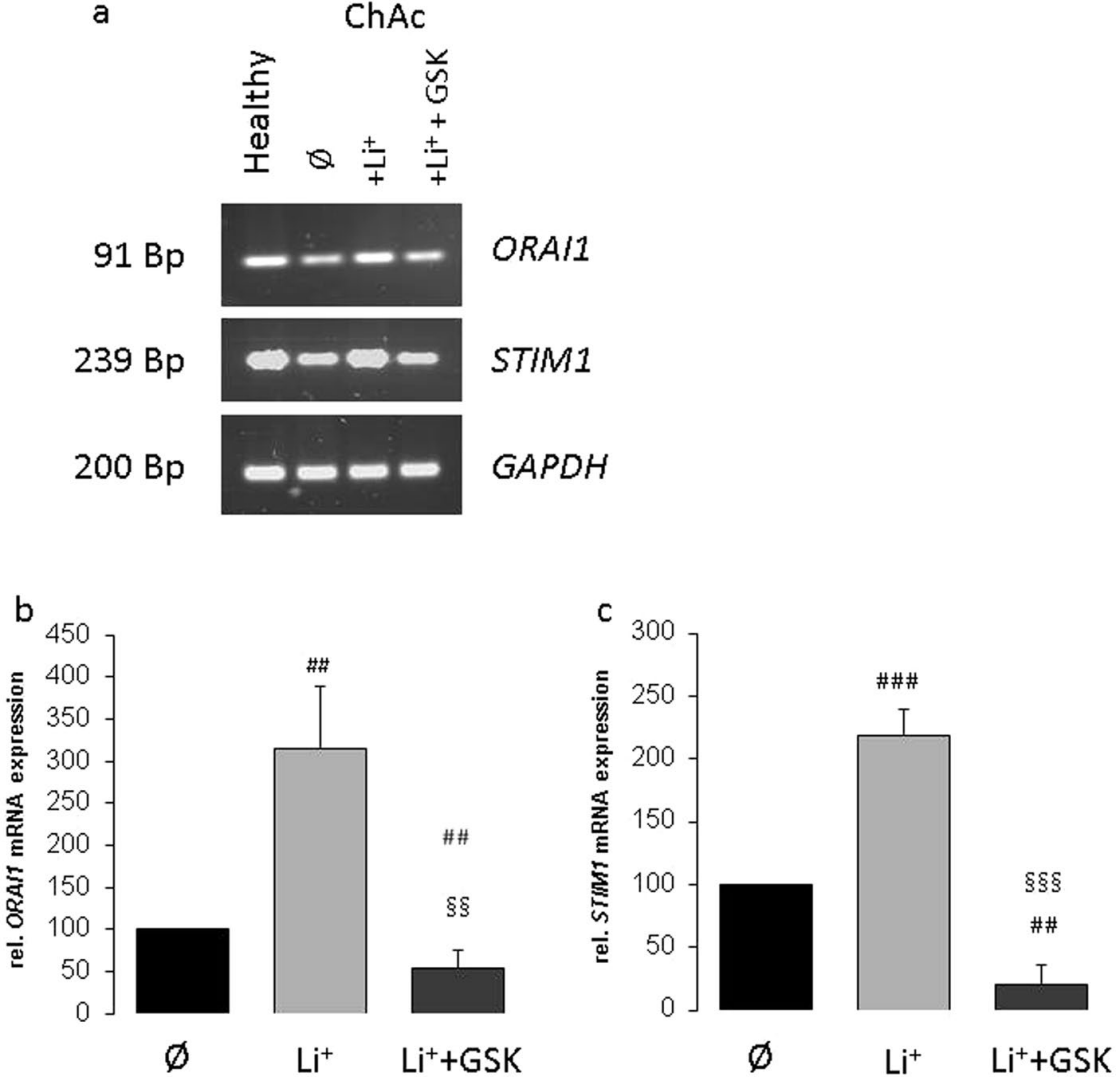
Effect of lithium treatment without and with SGK1 inhibitor GSK650394 on ORAI1 and STIM1 transcript levels in ChAc neurons. (**a**) Original gels illustrating specificity of PCR products. (**b**,**c**) Arithmetic means (±SEM, n = 5) of (**b**) ORAI1 and (**c**) STIM1 transcript levels in neurons generated from ChAc patients without (black bars) or with lithium (24 h, 2 mM) treatment without (light grey bar) and with (dark grey bar) presence of SGK1 inhibitor GSK650394 (10 µM). ^##^(p < 0.01), ^###^(p < 0.001) indicates statistically significant difference to respective value in the absence of lithium treatment. ^§§^(p < 0.01), ^§§§^(p < 0.001) indicates statistically significant difference to respective value in the absence of GSK650394.

In order to test whether transcription of ORAI1 and STIM1 in ChAc neurons is sensitive to lithium, ChAc neurons were incubated for 24 hours in the absence or presence of 2 mM lithium. The ORAI1 and STIM1 mRNA abundance was subsequently determined using qRT-PCR. As displayed in Fig. 2, ORAI1 and STIM1 transcript levels were in ChAc neurons significantly increased by treatment with 2 mM LiCl. The ORAI1 and STIM1 transcript levels were similar in lithium treated ChAc neurons and in untreated neurons from healthy volunteers. The effect of lithium on ORAI1 and STIM1 transcript levels was reversed by additional treatment with the serum & glucocorticoid inducible kinase (SGK1) inhibitor GSK650394 (10 µM). In the presence of lithium and GSK650394, the ORAI1 and STIM1 transcript levels were even significantly lower than the transcript levels of untreated ChAc neurons (Fig. 2)  ﻿(Complete gel picture in Supplementary Figure S2).

**Figure 3:**
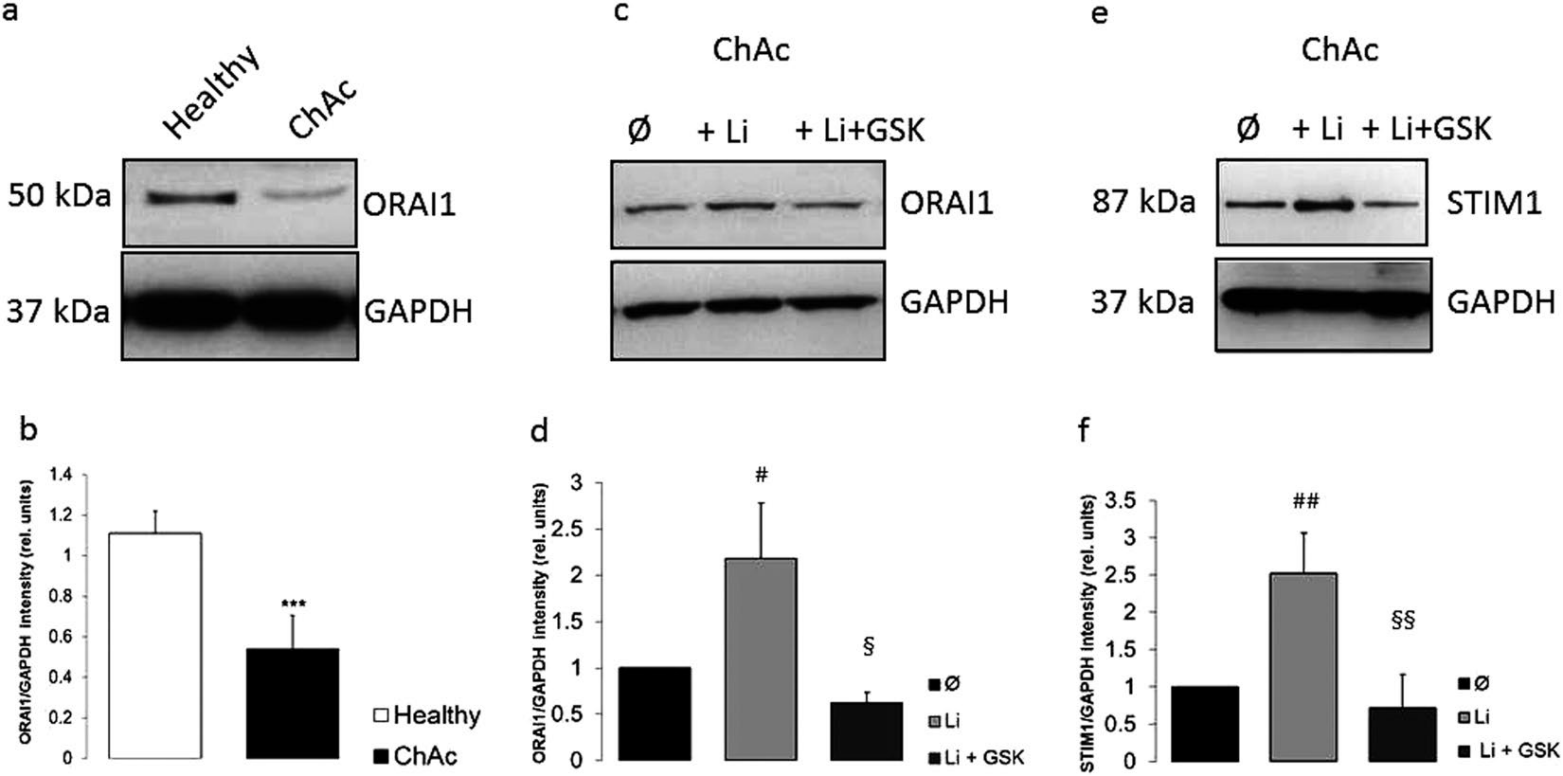
ORAI1 and STIM1 protein abundance in neurons generated from healthy volunteers and from ChAc patients without or with lithium treatment without and with SGK1 inhibitor GSK650394. (**a**) Original Western blot of ORAI1 protein abundance in neurons differentiated from healthy volunteers (healthy) and in neurons from ChAc patients (ChAc). (**b**) Arithmetic means (±SEM, n = 4) of ORAI1 protein levels in neurons generated from healthy volunteers (white bar) and in neurons from ChAc patients (black bar). (**c**) Original Western blot of ORAI1 protein abundance in neurons from ChAc patients without (Ø) and with prior lithium (24 h, 2 mM) treatment without (+Li) and with (+Li + GSK) additional presence of SGK1 inhibitor GSK650394 (10 µM). (**d**) Arithmetic means (±SEM, n = 4) of ORAI1 protein levels in neurons differentiated from ChAc patients without (black bar) or with lithium (24 h, 2 mM) treatment without (light grey bar) and with (dark grey bar) presence of SGK1 inhibitor GSK650394 (10 µM). (**e**) Original Western blot of STIM1 protein abundance in neurons from ChAc patients without (Ø) and with prior lithium (24 h, 2 mM) treatment without (+Li) and with (+Li + GSK) additional presence of SGK1 inhibitor GSK650394 (10 µM). (**f**) Arithmetic means (±SEM, n = 3) of STIM1 protein abundance in neurons differentiated from ChAc patients without (black bar) or with lithium (24 h, 2 mM) treatment without (light grey bar) and with (dark grey bar) presence of SGK1 inhibitor GSK650394 (10 µM). ***(p < 0.001) indicates statistically significant difference to respective value in neurons from healthy volunteers, ^#^(p < 0.05), ^##^(p < 0.01) indicates statistically significant difference to respective value in absence of lithium. ^§^(p < 0.05), ^§§^(p < 0.01) indicates statistically significant difference to respective value in the absence of GSK650394.

Western blotting was employed to test whether the differences of ORAI1 and STIM1 transcript levels between healthy individuals and ChAc patients were paralleled by similar differences in protein abundance. As illustrated in Fig. 3a,b, the ORAI1 and STIM1 protein abundance was significantly lower in ChAc neurons than in neurons from healthy volunteers. In line with the qRT-PCR results, ORAI1 and STIM1 protein abundance was significantly higher in ChAc neurons following a 24 hours incubation in the presence than in the absence of 2 mM lithium (Fig. 3c,d). Accordingly, lithium up-regulates ORAI1 and STIM1 protein expression. The ORAI1 and STIM1 protein abundance was in lithium treated ChAc neurons similarly high as in untreated neurons from healthy volunteers. The additional treatment with the SGK1 inhibitor GSK650394 (10 µM) decreased the ORAI1 and STIM1 protein abundance. In the presence of both, lithium and GSK650394, the ORAI1 and STIM1 protein abundance were not significantly different from the ORAI1 and STIM1 protein abundance in the absence of lithium and GSK650394 (Fig. 3)﻿﻿﻿ (Complete Western blot in Supplementary Figure S3 for ﻿ORAI﻿1 and Figure S4 for STIM1﻿).

### Store operated Ca^2+^ entry

**Figure 4:**
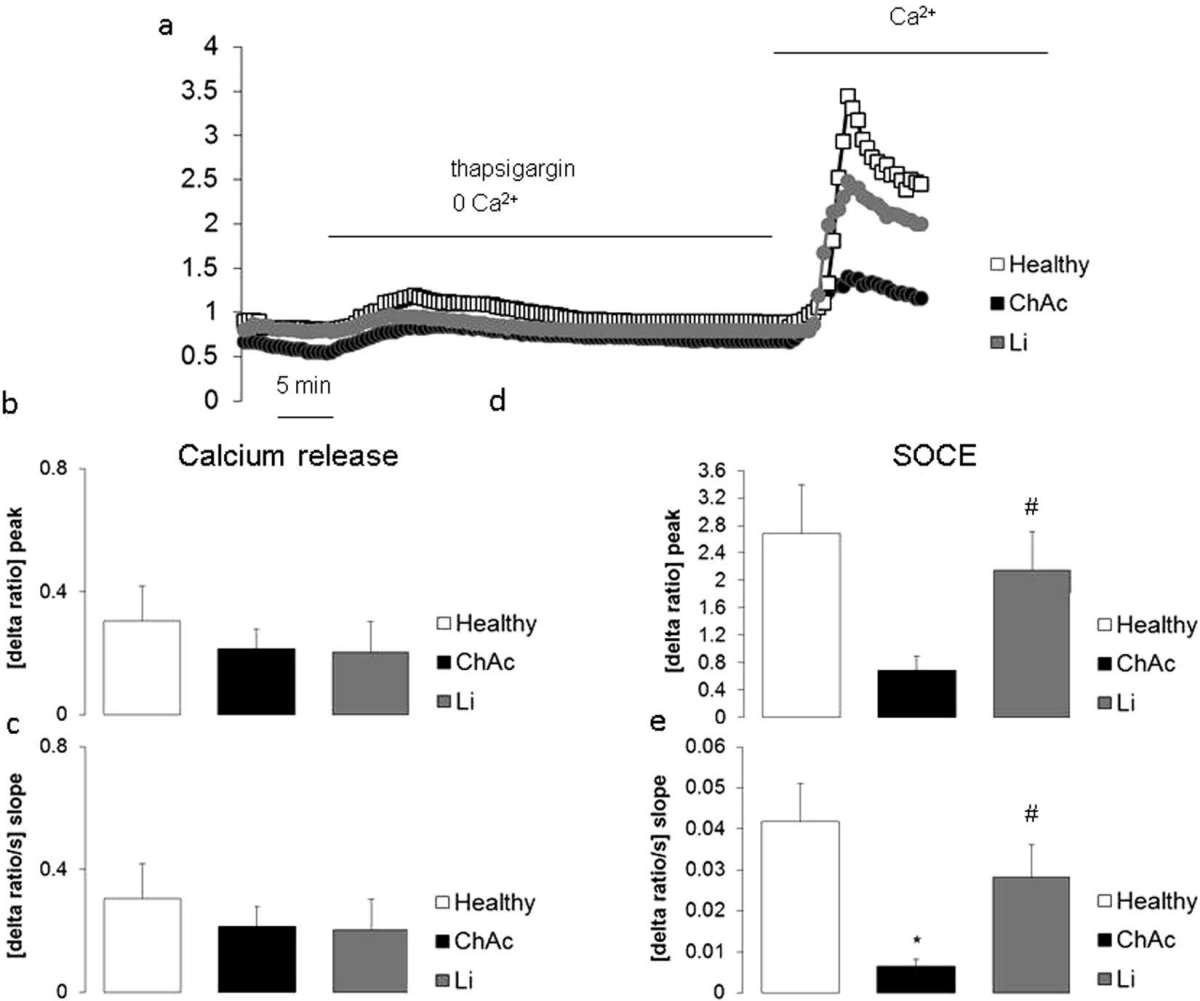
Intracellular Ca^2+^ release and store-operated Ca^2+^ entry (SOCE) in neurons from healthy volunteers and from ChAc patients without or with lithium treatment. (**a**) Representative tracings of Fura-2 fluorescence-ratio in fluorescence spectrometry before and following extracellular Ca^2+^ removal and addition of thapsigargin (1 µM), as well as re-addition of extracellular Ca^2+^ in neurons generated from healthy volunteers (white squares) and from ChAc patients without (black circles) and with (grey circles) lithium (24 h, 2 mM) treatment. (**b**,**c**). Arithmetic means (±SEM, n = 37–74 cells from 4 individuals) of slope (**b**) and peak (**c**) increase of fura-2-fluorescence-ratio following addition of thapsigargin (1 µM) in control neurons (white bar) and in ChAc neurons without (black bar) and with (grey bar) lithium (24 h, 2 mM) treatment. (**d**,**e**) Arithmetic means (±SEM, n = 37–74 cells from 4 individuals) of slope (**d**) and peak (**e**) increase of fura-2-fluorescence-ratio following re-addition of extracellular Ca^2+^ in neurons from healthy volunteers (white bars) and in neurons from ChAc patients without (black bar) and with (grey bar) lithium (24 h, 2 mM) treatment. *(p < 0.05) indicates statistically significant difference to respective value in neurons from healthy volunteers, ^#^(p < 0.05) indicates statistically significant difference to respective value in absence of lithium.

Decreased expression of ORAI1 and STIM1 were expected to be paralleled by impaired store operated Ca^2+^ entry (SOCE). Fura2 fluorescence was thus employed to quantify the cytosolic Ca^2+^ concentration ([Ca^2+^]_i_). For determination of SOCE, the intracellular stores were emptied by exposure of the cells to the sarco-/endoplasmic reticulum Ca^2+^-ATPase (SERCA) inhibitor thapsigargin (1 µM) in the absence of extracellular Ca^2+^. Re-addition of extracellular Ca^2+^ in the continued presence of thapsigargin resulted in a sharp increase of [Ca^2+^]_i_ reflecting SOCE. As shown in Fig. 4a–c, emptying the intracellular Ca^2+^ stores with thapsigargin was followed by a transient increase in [Ca^2+^]_i_ to similar values in control neurons and ChAc neurons. The increase of [Ca^2+^]_i_ following re-addition of extracellular Ca^2+^ in the continued presence of thapsigargin was significantly blunted in ChAc neurons as compared to control neurons (Fig. 4d,e). Both, slope and peak of [Ca^2+^]_i_ increase following re-addition of extracellular Ca^2+^ were significantly lower in ChAc neurons than in control neurons. SOCE was virtually abrogated by ORAI1 inhibitor 2-APB (50 µM) (data not shown).

Further experiments explored the effect of lithium on SOCE. To this end, ChAc neurons were incubated for 24 hours in the presence or absence of 2 mM lithium. As a result, without triggering SOCE, [Ca^2+^]_i_ was in ChAc neurons similar in the absence or presence of lithium (data not shown). Both, peak and slope of SOCE were, however, significantly higher in lithium treated than in untreated ChAc neurons (Fig. 4d,e). Following lithium treatment, SOCE was in ChAc neurons of similar magnitude as in untreated neurons from healthy volunteers.

### Apoptosis

**Figure 5:**
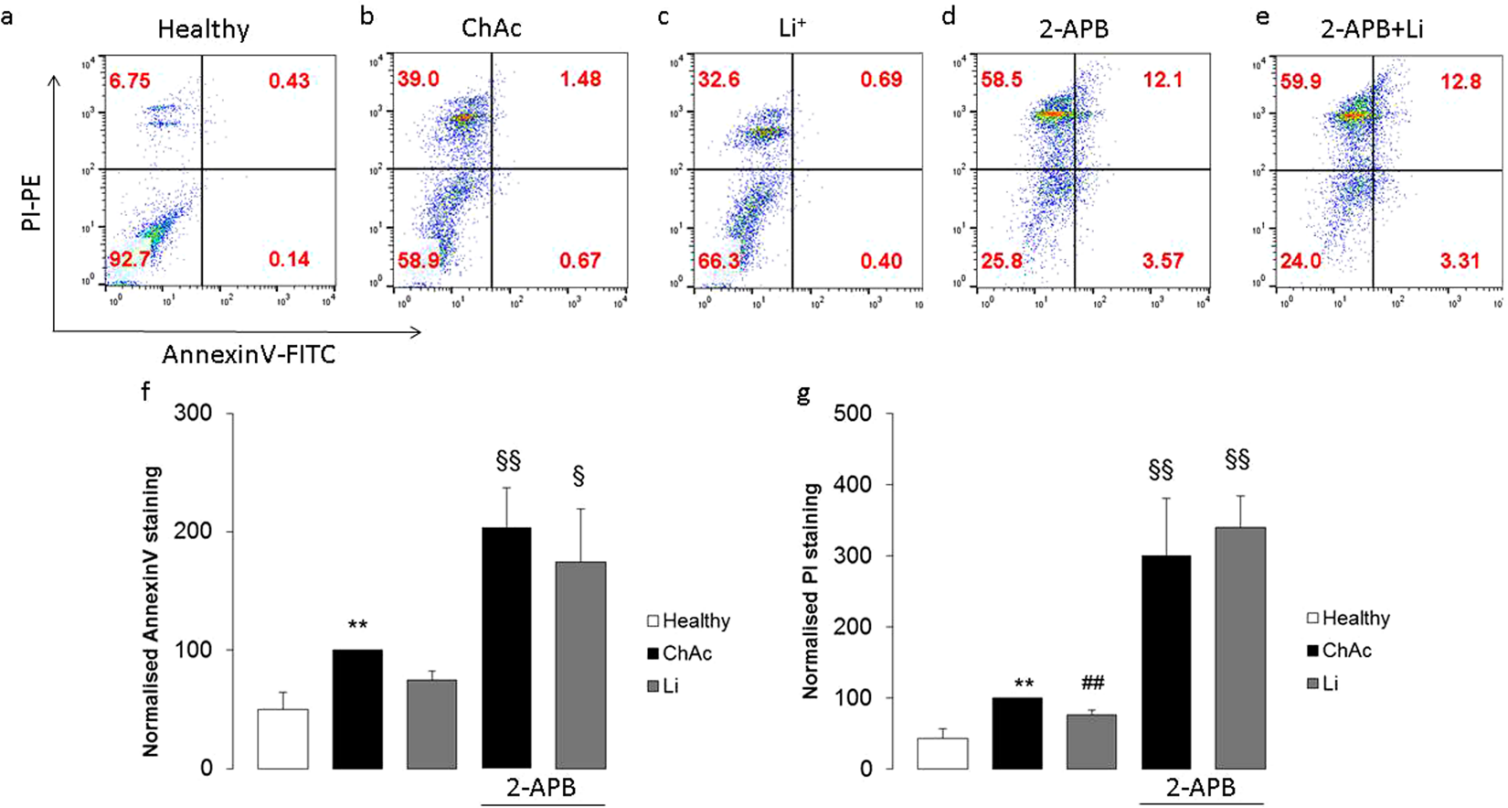
Phosphatidylserine translocation and propidium iodide uptake in neurons from healthy volunteers and from ChAc patients without and with treatment with lithium and/or 2-APB. (**a**–**e**) Representative dot blots of propidium iodide staining versus annexin-V-binding of neurons generated from (**a**) healthy volunteers and from (**b**–**e**) ChAc patients (**b**) without treatment and with lithium treatment (2 mM) alone (**c**), with 50 µM 2-APB alone (**d**), and with lithium and 2-APB together (**e**). **(f**,**g**) Arithmetic means (±SEM, n = 4–8 from 4 individuals) of the normalised (**f**) annexin-V-binding or (**g**) propidium iodide stained neurons from healthy volunteers (white bars) and from ChAc patients without (black bars) or with (grey bars) 2 mM lithium treatment without (left bars) and with (right bars) presence of 50 µM 2-APB (2-APB). **(p < 0.01) indicates statistically significant difference to respective value in neurons from healthy volunteers, ^##^(p < 0.01) indicates statistically significant difference to respective value in absence of lithium, ^§^(p < 0.05), ^§§^(p < 0.01) indicates statistically significant difference to respective value in absence of 2-APB (ANOVA).

Flow cytometry was employed to explore whether the differences in SOCE were paralleled by similar differences in apoptotic neuronal death. Apoptotic neurons were identified by measurement of annexin-V-binding and propidium iodide uptake. As apparent from Fig. 5a,b, the percentage of propidium iodide harboring and annexin-V-binding cells was significantly higher in ChAc neurons than in control neurons.

The percentage of propidium iodide harboring and annexin-V-binding ChAc neurons was significantly lower following a 24 hours incubation in the presence than in the absence of lithium (Fig. 5b,c). Exposure of ChAc neurons with ORAI1 blocker 2-APB (50 µM) dramatically and significantly increased the percentage of propidium iodide harboring and annexin-V-binding cells and virtually abrogated the effect of lithium on the percentage of propidium iodide harboring and annexin-V-binding ChAc neurons (Fig. 5d,e).

## Discussion

The present study uncovers a novel pathophysiological mechanism in chorea-acanthocytosis, i.e. deranged regulation of ORAI1 and STIM1 expression with subsequent impairment of store operated Ca^2+^ entry (SOCE). SOCE is significantly and markedly down-regulated in neurons lacking functional chorein.

SOCE may trigger oscillations of cytosolic Ca^2+^ activity ([Ca^2+^]_i_)^28^, resulting from rapid Ca^2+^ entry due to activation of SOCE followed by inhibition of ORAI1 and rapid decrease of [Ca^2+^]_i_ due to subsequent Ca^2+^ extrusion^29^. The repetitive short pulses of [Ca^2+^]_i_ activate Ca^2+^ dependent transcription factors and reorganize the actin filament network without triggering the detrimental consequences of sustained increases of [Ca^2+^]_i_^30, 31^.

The Ca^2+^ oscillations influence diverse cellular functions^32, 33^ including entry into the S and the M phase of the cell cycle^34^ and cell survival^35, 36^. Along those lines the ORAI isoforms^19^, and their regulators STIM 1 or 2^20^ contribute to the orchestration of survival, proliferation, and migration of tumor cells^37–40^ and neural stem/progenitor cells^41^. As shown here, pharmacological inhibition of ORAI1 triggers apoptosis and disrupts the supporting effect of lithium on neuronal cell survival.

In contrast to Ca^2+^ oscillations, sustained increases of cytosolic Ca^2+^ activity have been shown to stimulate apoptosis in a variety of cell types^42–44^. Thus, cell survival or death depends on a delicate balance between stimulators and inhibitors of Ca^2+^ entry.

The present study sheds some light on the signalling linking chorein deficiency and ORAI1 protein abundance. Previous observations in other cell types revealed that ORAI1 expression is up-regulated by the PI3K dependent^45^ serum & glucocorticoid inducible kinase SGK1^46, 47^. Along those lines, the effect of lithium is reversed in the presence of SGK1 inhibitor GSK650394. SGK1 is effective by NFκB dependent up-regulation of ORAI1 expression and by inhibition of Nedd4-2 triggered degradation of ORAI1 protein^46, 47^. Chorein deficiency impairs activation of PI3K^1–3^ and is thus expected to disrupt PI3K/SGK1/ NFκB dependent ORAI1 upregulation. The observed signalling may thus well contribute to the anti-apoptotic effect of PI3K which confers survival of a wide variety of cells including cancer cells^48–51^ and neurons^52–55^.

The present observations further uncover an anti-apoptotic effect of lithium in neurons from chorea-acanthocytosis. The effect of lithium apparently requires up-regulation of SOCE. Lithium has previously been shown to counteract neurodegeneration in several diseases such as Huntington’s chorea, Alzheimer’s disease, Parkinson’s disease, amyotrophic lateral sclerosis as well as spinocerebellar ataxias type 1 and type 3^25–27, 56, 57^. Mechanisms involved in the neuroprotective effect of lithium include direct or Akt-mediated inhibition of glycogen synthase kinase GSK-3β, Akt-mediated inhibition of the proapoptotic forkhead box class O transcription factor Foxo3a and murine double minute (MDM), stimulation of production and activity of neuroprotective brain derived neurotrophic factor BDNF, up-regulation of antiapoptotic protein Bcl-2, as well as down-regulation of proapoptotic transcription factor p53, of the proapoptotic proteins Bad and Bax, of glutamate excitotoxicity, of calpain and of oxidative stress^27, 58^. We show here that the protective effect of lithium is abrogated by Orai1 inhibition. In view of the present observations, it is tempting to speculate that lithium may modify the course of neurodegeneration in part by up-regulating neuronal ORAI1 expression and SOCE thus stimulating proliferation of neuronal progenitor cells and inhibiting neuronal apoptosis.

In view of the present observations clinical studies appear warranted exploring the effect of lithium treatment on the clinical course of chorea-acanthocytosis. To the extent that neuronal apoptosis is the decisive pathophysiological mechanism leading to this devastating disease, lithium treatment may delay or even halt the deterioration of neuronal function in affected patients.

In conclusion, lack of Chorein in chorea-acanthocytosis downregulates ORAI1 expression and store operated Ca^2+^ entry leading to compromised neuronal cell survival. Conversely, lithium up-regulates store operated Ca^2+^ entry and attenuates neuronal apoptosis, an effect abrogated by pharmacological inhibition of ORAI1.

## Methods

### Generation of iPSCs

The study has been approved by the Ethical Commission of the University of Tübingen (598/2011). Informed consent was obtained from all participants and/or their legal guardian/s. Human dermal fibroblasts were isolated from ChAc patients (n = 2) and healthy volunteers (n = 3). We confirm that all methods performed, including obtaining of consent, were performed in accordance with the relevant guidelines and regulations as approved by the ethics committee. Dermal fibroblasts were cultivated in fibroblast medium, consisting of DMEM (Biochrom, Berlin, Germany) supplemented with 10% fetal calf serum (FCS, Life technologies, Thermo Fisher Scientific, Waltham, Massachusetts) and 1% L-Glutamine (Biochrom). Induced pluripotent stem cells (iPSCs) were generated following a protocol published previously^59^, with minor modifications. In brief, 1 × 10^5^ fibroblasts were electroporated (Nucleofector 2D, Lonza) with a total of 1 μg per plasmid carrying the sequences for hOCT4, hSOX2, hKLF4, hL-MYC and hLIN28. After cultivation in fibroblast medium for 1 day, 2 ng/ml FGF-2 (Peprotech) was supplemented to the medium. From day 3 on, cells were cultivated in Essential 8 (E8) medium containing 100 µM NaB (Sigma-Aldrich). iPSC colonies were picked manually after 3–4 weeks and further expanded in Matrigel coated 6-well plates. At passage 7–10, iPSCs were characterized and frozen in E8 medium supplemented with 40% KOSR (Thermo Fisher Scientific), 10% DMSO (Sigma-Aldrich), and 1 µM Y-27632 (Selleckchem, Munich, Germany). Characterization of generated iPSCs included genomic validation via exclusion of plasmid-integration, SNParray analysis for genetic integrity, and resequencing of mutation site, as well as functional validation via confirmation of expression of pluripotency marker, and verification of the *in vitro* differentiation potential as described previously^60^.

### Differentiation of iPSCs to neurons

Cortical neurons were generated as described previously^61^. Briefly, neural induction of iPSCs was achieved by addition of dual SMAD inhibitors (10 µM SB431542 (Sigma-Aldrich) and 500 nM LDN-193189 (Sigma-Aldrich)) to 3N medium. Cells were collected at day 10 and further expanded by cultivation in 3N medium supplemented with 20 ng/ml FGF-2 for 2 days. From day 12 on, cells were cultivated in 3N medium with medium change every other day. Cell cultures were passaged at day 27 and replated appropriately for the specific assay (RNA/Protein isolation: 5 × 10^5^ cells per cm^2^; FACS analysis: 2.5 × 10^5^ per cm^2^; Ca^2+^ measurements: 5 × 10^4^ per cm^2^). Where indicated, 2 mM lithium (Sigma-Aldrich) was added to the medium 24 hours prior to measurements. Analysis was performed between day 37 and 41.

### Quantitative PCR

Transcript levels of ORAI1, STIM1, STIM2, and house keeping Gapdh were determined by RT-PCR as described previously^23, 62^. Total RNA was extracted in TriFast (Peqlab, Erlangen, Germany) according to the manufacturer’s instructions. After DNAse digestion reverse transcription of 2 µg RNA was performed using random hexamers (Roche Diagnostics, Penzberg, Germany) and SuperScriptIII reverse transcriptase (Invitrogen, Carlsbad, CA, USA). Real-time polymerase chain reaction (RT-PCR) amplification of the respective genes were set up in a total volume of 20 µl using 40 ng of cDNA, 500 nM forward and reverse primer and 2x GoTaq® qPCR Master Mix (Promega, Hilden, Germany) according to the manufacturer’s protocol. Cycling conditions were as follows: initial denaturation at 95 °C for 2 minutes, followed by 40 cycles of 95 °C for 15 seconds, 55 °C for 15 seconds and 68 °C for 20 seconds. For amplification the following primers were used (5′->3′orientation):

GAPDH:

fw: TGAGTACGTCGTGGAGTCCAC;

rev: GTGCTAAGCAGTTGGTGGTG

ORAI11:

fw: CGTATCTAGAATGCATCCGGAGCC;

rev: CAGCCACTATGCCTAGGTCGACTAGC

STIM1:

fow: CCTCGGTACCATCCATGTTGTAGCA

rev: GCGAAAGCTTACGCTAAAATGGTGTCT

STIM2:

for: CAAGTTGCCCTGCGCTTTAT

rev: ATTCACTTTTGCACGCACCG

Specificity of PCR products was confirmed by analysis of a melting curve. Real-time PCR amplifications were performed on a CFX96 Real-Time System (Bio-Rad, Munich, Germany) and all experiments were done in duplicate. The house-keeping gene Glyceraldehyde 3-phosphate dehydrogenase (GAPDH) was amplified to standardize the amount of sample RNA.

### Western Blotting

Protein abundance of ORAI1, STIM1 and GAPDH was determined by Western blotting as described previously^23, 62^. To this end, cells were centrifuged for 5 minutes at 240 g and 4 °C. The pellet was washed twice with ice cold PBS and suspended in 200 μl ice-cold RIPA lysis buffer (Thermo Fisher Scientific, USA) containing Halt Protease and Halt Phosphatase Inhibitor Cocktail (Thermo Fisher Scientific, USA). Protein concentration was determined using the Bradford assay (BioRad, München, Germany). 100 µg of protein were solubilized in sample buffer at 95 °C for 5 min. The proteins were separated by a 10% SDS-PAGE in a Glycine-Tris buffer and electro-transferred onto nitrocellulose membranes for 70 min. After blocking with 5% milk in TBST at room temperature for 1 h, the membranes were incubated with primary anti-ORAI1 antibody (1:1000, Proteintec), anti STIM1 antibody (1:2000, Cell Signaling) and anti-GAPDH antibody (1:2000, Cell Signaling) at 4 °C overnight. After washing (TBST), the blots were incubated with secondary anti-rabbit antibody conjugated with horseradish peroxidase (1:2000, Cell Signaling) for 1 h at room temperature. Protein bands were detected after additional washes (TBST) with an ECL detection reagent (Amersham, Freiburg, Germany) and quantified with Quantity One Software (BioRad, München, Germany). To assign the right protein size we used Protein-Marker VI (Peqlab, Erlangen, Germany).

### Ca^2+^ measurements

Fura-2 fluorescence was taken as a measure of cytosolic Ca^2+^ concentration ([Ca^2+^]_i_), as described previously^23, 62^, For this purpose cells were loaded with Fura-2/AM (2 µM, Invitrogen, Goettingen, Germany) for 20 min at 37 °C. Cells were excited alternatively at 340 nm and 380 nm through an objective (Fluor 40×/1.30 oil) built in an inverted fluorescence microscope (Axiovert 100, Zeiss, Oberkochen, Germany). Emitted fluorescence intensity was recorded at 505 nm. Data were acquired using specialized computer software (Metafluor, Universal Imaging, Downingtown, USA). Cytosolic Ca^2+^ activity was estimated from the 340 nm/380 nm ratio. SOCE was determined by extracellular Ca^2+^ removal and subsequent Ca^2+^ re-addition in the presence of thapsigargin (1 µM, Invitrogen). For quantification of Ca^2+^ entry, the slope (delta ratio/s) and peak (delta ratio) were calculated following re-addition of Ca^2+^.

Experiments were performed with Ringer solution containing (in mM): 125 NaCl, 5 KCl, 1.2 MgSO_4_, 2 CaCl_2_, 2 Na_2_HPO_4_, 32 HEPES, 5 glucose, pH 7.4. To reach nominally Ca^2+^-free conditions, experiments were performed using Ca^2+^-free Ringer solution containing (in mM): 125 NaCl, 5 KCl, 1.2 MgSO_4_, 2 Na_2_HPO_4_, 32 HEPES, 0.5 EGTA, 5 glucose, pH 7.4.

### Analysis of apoptosis

Propidium iodide (PI, Sigma-Aldrich) uptake was taken as a measure of cell membrane permeability and annexin V-FITC (Immunotools, Friesoythe, Germany) binding as a measure of cell membrane scrambling with phosphatidylserine translocation at the cell surface, as described previously^63, 64^. To this end, cells were incubated in 100 µl Ringer solution containing 5 mM Ca^2+^ and 1 µl annexin V-FITC After incubation, the cells were centrifuged at 1000 r.p.m. for 5 minutes. Then, cells were stained with annexin V-FITC (Immunotools, Friesoythe, Germany) to assess phosphatidylserine exposure and propidium iodide (PI) to estimate cell membrane integrity. To this end, cells were incubated in 100 µl Ringer solution containing 5 mM Ca^2+^ and 1 µl annexin V-FITC (Immunotools, Friesoythe, Germany) and 0.075 µl PI stock solution (25 mg/ml PI in PBS) for 15 min at 37 °C. Then, 100 µl of Ringer solution were added and annexin V-FITC as well as PI fluorescence were determined by flow cytometry using a FACS calibur (BD, Heidelberg, Germany).

### Statistics

Data are expressed as arithmetic means ± SEM. Statistical analysis was made by unpaired t-test or Mann-Whitney test or analysis of variance (ANOVA), as appropriate. p < 0.05 was considered as statistically significant.

## Electronic supplementary material

Supplementary Information
