# Supplementary material for: Lithium Sensitive ORAI1 Expression, Store Operated Ca2+ Entry and Suicidal Death of Neurons in Chorea-Acanthocytosis
Source: Sci Rep. 2017 Jul 25;7:6457. doi: 10.1038/s41598-017-06451-1 (PMC5526875; doi:10.1038/s41598-017-06451-1)
Supplement: Supplementary file 1 — Supplementary Information [file 41598_2017_6451_MOESM1_ESM.doc]

**Lithium Sensitive Orai1 Expression, Store Operated Ca^2+^ Entry and Suicidal Death of Neurons in Chorea-Acanthocytosis**

Lisann Pelzl^1^, Stefan Hauser^2^, Bhaeldin Elsir^1^, Basma Sukkar^1^, Itishri Sahu^1^, Yogesh Singh^1^, Philip Höflinger^2^, Rosi Bissinger^1^, Mohamed Jemaà^1^, Christos Stournaras^1,3^, Ludger Schöls^2,4#^, Florian Lang^1#^*

^#^Contributed equally and thus share last authorship

^1^Department of Internal Medicine III, University of Tübingen, Germany, ^2^German Center for Neurodegenerative Diseases, Tübingen, Germany, ^3^Department of Biochemistry, University of Crete Medical School, Heraklion, Greece, ^4^Department of Neurology and Hertie Institute for Clinical Brain Research, University of Tübingen, Germany

***Correspondence to**:

Prof. Dr. Florian Lang

Physiologisches Institut, Universität Tübingen

Gmelinstr. 5, D-72076 TÜBINGEN

Tel: +49 7071 29 72194, Fax: +49 7071 29 5618

e-mail: [florian.lang@uni-tuebingen.de](mailto:florian.lang@uni-tuebingen.de)


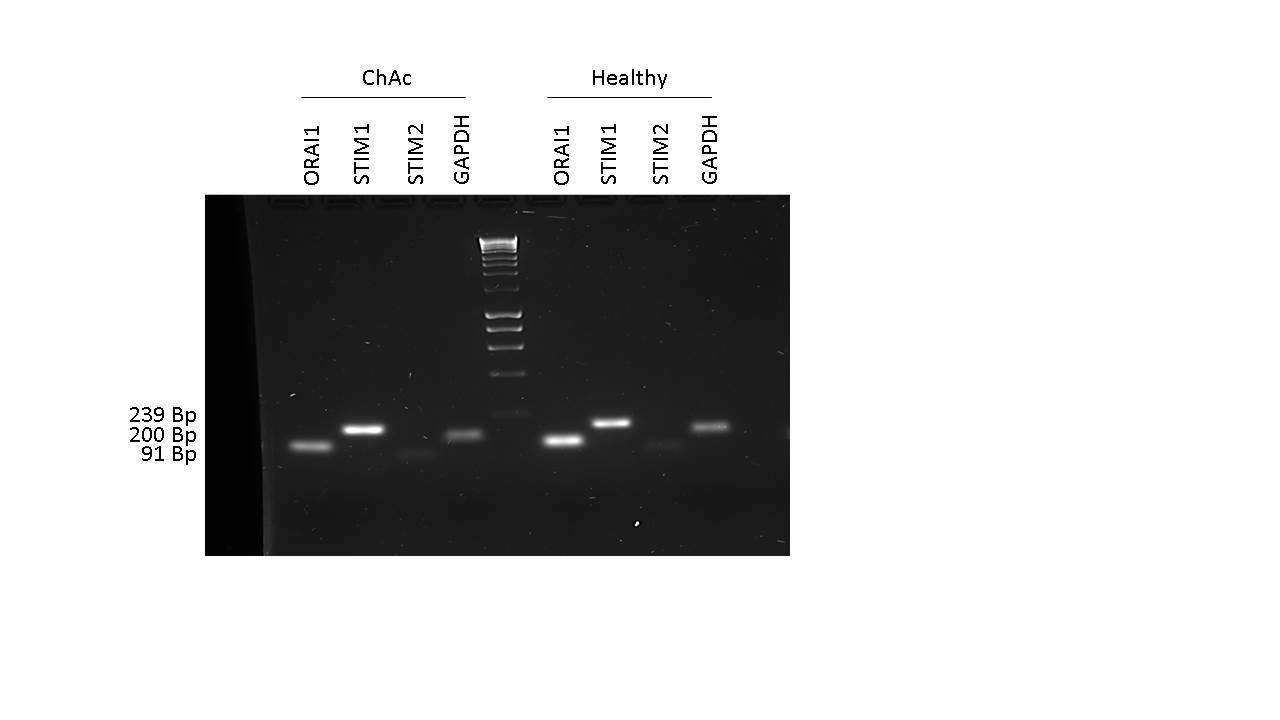
Supplementary Information

S1 : Orignial Gel for Figure 1

S2 : Orignial Gel for Figure
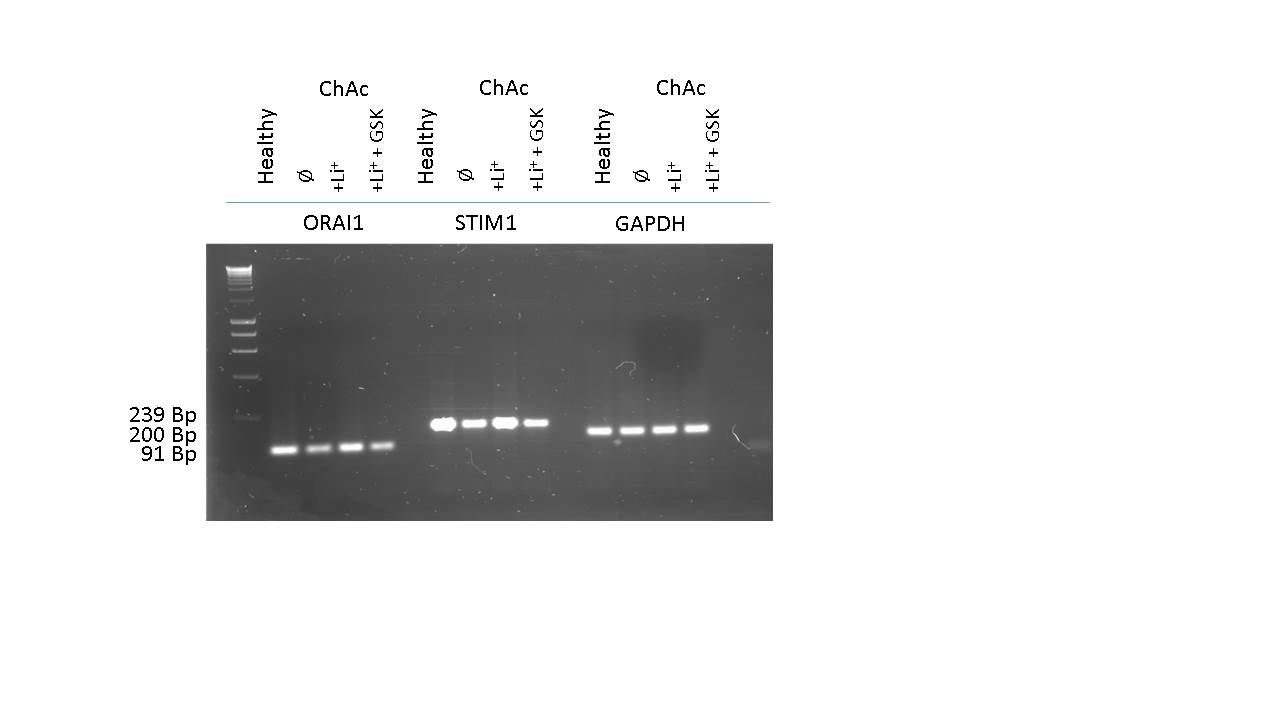
2

S3 : Orignial Western image for Figure
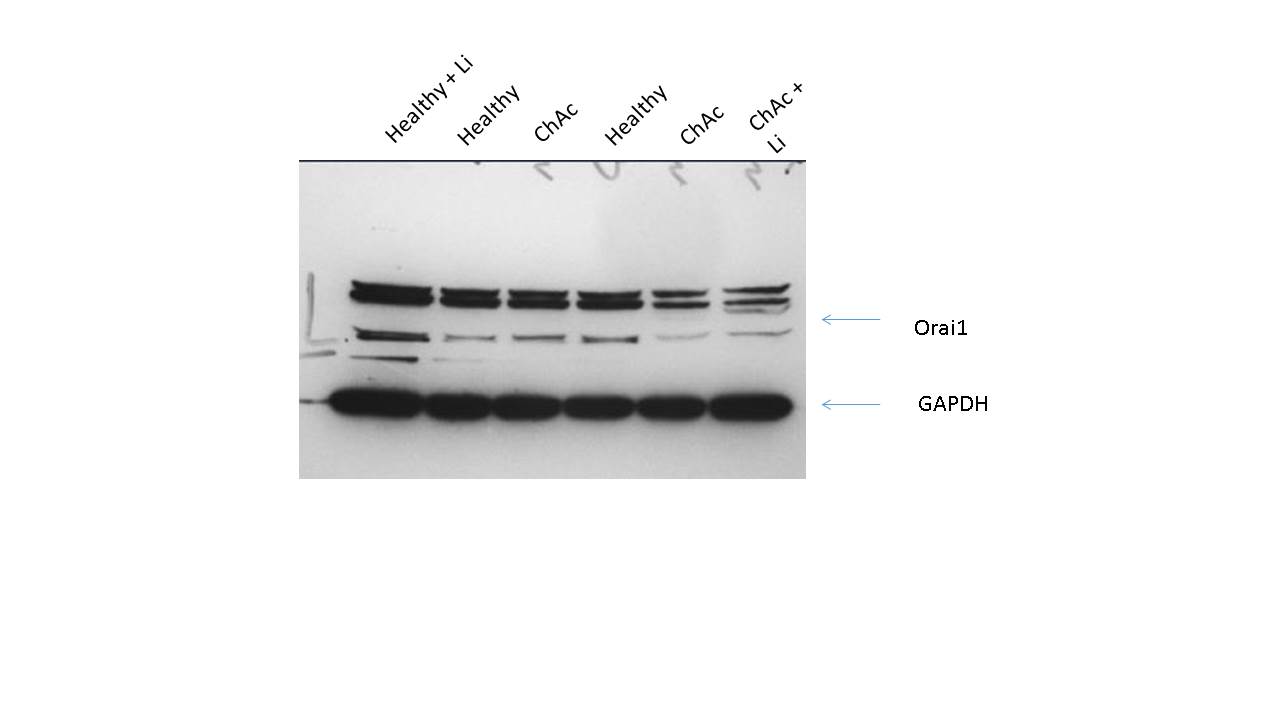
3

S4 : Orignial Western image for Figure
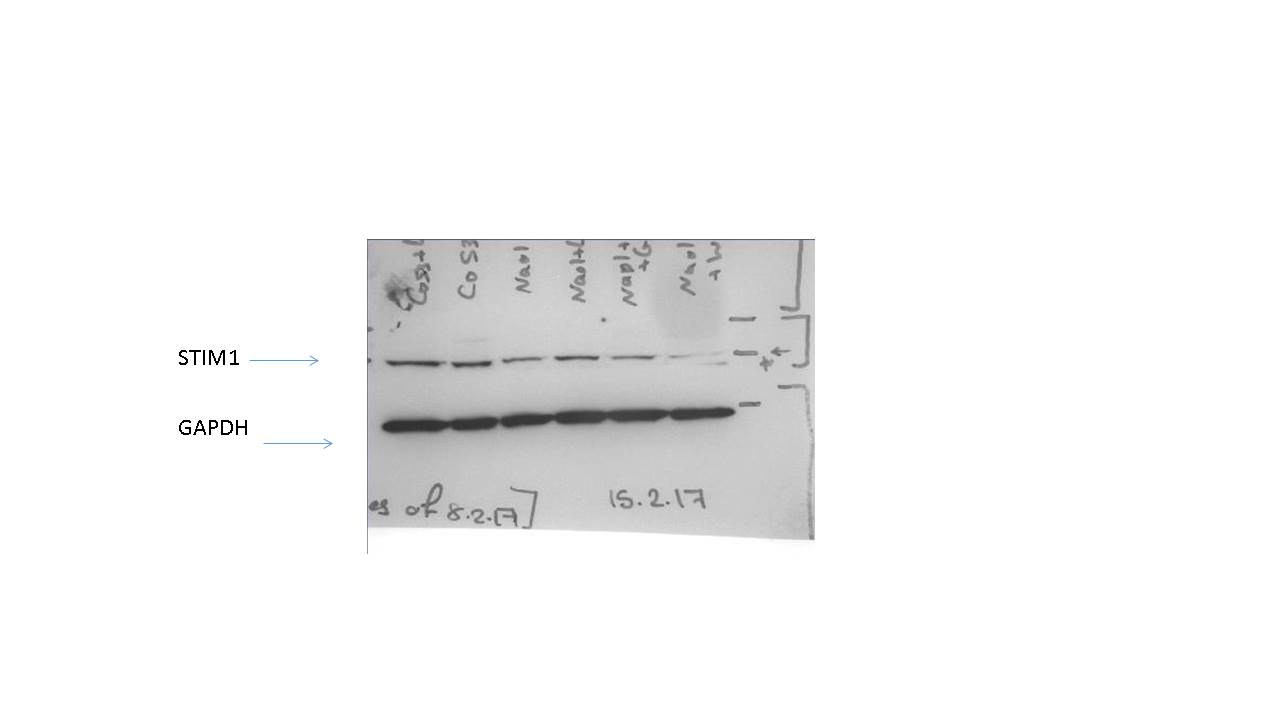
3
